# Exploratory PET/CT Radiomics for Predicting Early Progression in Locally Advanced Pancreatic Cancer

**DOI:** 10.3390/diagnostics16101499

**Published:** 2026-05-14

**Authors:** Michele Fiore, Ermanno Cordelli, Gian Marco Petrianni, Gabriele D’Ercole, Pasquale Trecca, Silvia Taralli, Vincenzo La Vaccara, Damiano Caputo, Edy Ippolito, Maria Lucia Calcagni, Paolo Soda, Sara Ramella

**Affiliations:** 1 Research Unit of Radiation Oncology, Department of Medicine and Surgery, Università Campus Bio-Medico di Roma, Via Alvaro del Portillo, 21, 00128 Roma, Italy; m.fiore@unicampus.it (M.F.); e.ippolito@unicampus.it (E.I.); s.ramella@unicampus.it (S.R.); 2 Operative Research Unit of Radiation Oncology, Fondazione Policlinico Universitario Campus Bio-Medico, Via Alvaro del Portillo, 200, 00128 Roma, Italy; g.dercole@policlinicocampus.it (G.D.); p.trecca@policlinicocampus.it (P.T.); 3 Institute of Nanotechnology (NANOTEC), National Research Council, c/o Campus EcoTekne, Via Monteroni, 73100 Lecce, Italy; e.cordelli@unicampus.it; 4 Department of Experimental Medicine, Università del Salento, Via Monteroni, 73100 Lecce, Italy; 5 Nuclear Medicine Unit, Diagnostic Imaging and Radiation Oncology Department, Fondazione Policlinico Universitario A. Gemelli IRCCS, Largo Francesco Vito 1, 00168 Rome, Italy; silvia.taralli@policlinicogemelli.it (S.T.); marialucia.calcagni@unicatt.it (M.L.C.); 6 Operative Research Unit of General Surgery, Fondazione Policlinico Universitario Campus Bio-Medico, Via Alvaro del Portillo, 200, 00128 Roma, Italy; v.lavaccara@policlinicocampus.it (V.L.V.); d.caputo@unicampus.it (D.C.); 7 Research Unit of General Surgery, Università Campus Bio-Medico di Roma, Via Alvaro del Portillo, 21, 00128 Roma, Italy; 8 Nuclear Medicine Institute, Department of Radiological and Hematological Sciences, Università Cattolica del Sacro Cuore, 00168 Rome, Italy; 9 Unit of Computer Systems and Bioinformatics, Department of Engineering, Università Campus Bio-Medico di Roma, Via Alvaro del Portillo 21, 00128 Rome, Italy; p.soda@unicampus.it; 10 Department of Diagnostics and Intervention, Radiation Physics, Biomedical Engineering, Umeå University, Universitetstorget 4, 901 87 Umeå, Sweden

**Keywords:** pancreatic cancer, radiomics, PET/CT

## Abstract

**Background/Objectives:** Early progression (EP) occurs in a subset of patients with locally advanced pancreatic cancer (LAPC), limiting the clinical benefit of treatment, and it remains difficult to predict. **Methods:** We developed a multiparametric predictive model integrating baseline ^18^F-FDG PET/CT radiomic features with clinical and biological data. A total of 242 radiomic features were extracted from each imaging modality (CT and PET), including first-order, gray-level co-occurrence matrix (GLCM), and local binary pattern (LBP-TOP) features, and combined with PET-derived metrics and clinical variables. Model development included cross-validation procedures and rigorous feature selection, followed by the training of a two-level decision tree classifier. **Results:** The model achieved an accuracy of 80.7% and an area under the curve (AUC) of 0.83. Integrated analysis of CT and PET texture enabled the identification of patients at high risk of EP prior to treatment initiation. **Conclusions:** PET/CT-based radiomic biomarkers, combined with clinical data, can non-invasively capture tumor heterogeneity and improve risk stratification in LAPC, supporting more personalized therapeutic decision-making.

## 1. Introduction

Pancreatic ductal adenocarcinoma (PDAC) is one of the most aggressive solid tumors, with a five-year survival rate below 10% and limited therapeutic options. It is currently the fourth most common cause of cancer-related death among men and the third among women. In 2024, an estimated 66,440 new cases were reported in the United States, of which 34,530 occurred in men and 31,910 in women. The estimated number of deaths was 51,750, including 27,270 men and 24,480 women [1]. At diagnosis, approximately 30% of patients present with locally advanced pancreatic cancer (LAPC), which is characterized by significant vascular involvement that precludes immediate surgical resection. Current clinical guidelines recommend induction chemotherapy followed by chemoradiation (CRT) as a potentially curative strategy for these patients or as a bridge to surgical resection in cases of borderline resectable disease [2]. However, despite therapeutic intensification, a subset of LAPC patients experience early progression (EP) of the disease within the first three months of treatment, resulting in treatment failure and poor outcomes. Early identification of these patients would be clinically valuable, as it would allow for a more individualized approach, either by modifying treatment intensity or by directing patients towards clinical trials or palliative care [3,4]. In recent years, advancements in imaging technology have improved disease assessment and management. Multidetector computed tomography (CT) with 3D reconstruction remains the gold standard for evaluating resectability and local tumor extension. Meanwhile, 18F-fluorodeoxyglucose positron emission tomography/computed tomography (^18^F-FDG PET/CT) has gained importance in detecting occult distant metastases, assessing treatment response, and guiding radiotherapy planning [5]. The role of ^18^F-FDG PET/CT is primarily complementary, providing additional metabolic information that cannot be captured by anatomical imaging alone. However, no clear superiority over contrast-enhanced CT has been established for primary diagnosis, and its clinical value lies mainly in prognostic stratification and treatment planning. Notably, PET/CT-derived metabolic data can refine target volume delineation by identifying subregions of a tumor that could benefit from dose escalation [6]. Beyond its role in staging and treatment planning, PET/CT has shown emerging prognostic value in pancreatic cancer. Previous studies have suggested that PET/CT semiquantitative parameters, such as the standardized uptake value (SUVmax), metabolic tumor volume (MTV), and total lesion glycolysis (TLG), can offer prognostic information when used alone or in combination with clinical biomarkers [7]. In a previous analysis from our institution, for instance, a CA 19-9 (carbohydrate antigen 19-9) level above 698 U/mL was the most predictive marker of EP (OR, 5.96; PPV, 61%), while TLG was the best predictor of local progression (OR, 9.75; PPV, 83%) and MTV correlated with overall survival (OR, 3.12; PPV, 88%). Combining these variables significantly increased the positive predictive value to above 90%, thereby reinforcing the potential roles of integrated imaging and clinical data in patient stratification [8]. Nevertheless, current prognostic models for LAPC, which rely on standard imaging parameters and a limited set of clinical biomarkers, suffer from suboptimal predictive accuracy, poor generalizability, and an inability to capture the full spectrum of tumor heterogeneity. Consequently, their ability to identify high-risk patients prior to treatment remains limited. This methodological shortcoming highlights the need for more comprehensive, multiparametric approaches that can exploit the rich, high-dimensional information embedded in baseline PET/CT images. Building on this concept of quantitative imaging, radiomics has emerged as a promising extension of conventional imaging analysis, aiming to extract a much broader range of data beyond traditional semiquantitative PET parameters. Radiomics is a term rooted in the field of ‘omics’ sciences. It refers to the extraction of large amounts of quantitative data from medical images using advanced mathematical and computational techniques. Like genomics and proteomics, the aim of radiomics is to identify imaging biomarkers that correlate with specific biological processes or pathological states. This approach provides insight into tumor behavior and enables the development of diagnostic, prognostic and predictive models [9]. As the omics discipline is most closely aligned with radiology, radiomics transforms standard medical imaging into a wealth of quantitative features that support clinical decision-making. Traditional imaging assessment, which is based on macroscopic tumor characteristics such as size, shape, or glucose uptake, offers only a limited perspective and often fails to capture the full biological complexity of solid tumors [10]. This is particularly true of PDAC, which exhibits remarkable spatial and temporal heterogeneity, both within a single lesion (intra-tumoral) and between different patients (inter-tumoral). This heterogeneity spans multiple levels, from genes and proteins to cells, the microenvironment, tissues, and organs. While conventional molecular analyses based on biopsy samples are limited by their invasive nature and sampling constraints, medical imaging provides a unique, non-invasive opportunity to characterize entire tumors and their heterogeneity [11]. This approach may enhance our ability to stratify patients and personalize therapeutic strategies. More recently, fibroblast activation protein inhibitor (FAPI)-based PET imaging has emerged as a promising alternative to FDG PET, particularly for pancreatic cancer, due to its lower background uptake and higher tumor-to-background contrast. Although early studies suggest potential advantages, radiomics applications based on FAPI PET remain limited, and comparative evidence with FDG-based radiomics is still lacking.

This study addresses the central question of whether integrating radiomic features from baseline PET/CT scans with clinical and biological data assessed prior to treatment can identify LAPC patients at high risk of early progression, thereby enabling alternative or intensified therapeutic strategies to be selected.

## 2. Materials and Methods

### 2.1. Study Population

A secondary analysis was conducted on a cohort of 57 patients with LAPC who were treated at our institution between July 2013 and March 2022 as part of two prospective clinical protocols. All patients were enrolled in a treatment program consisting of induction chemotherapy followed by curative-intent CRT. Baseline ^18^F-FDG PET/CT scans were performed at a single PET/CT center and were available for all included cases. Patients were eligible if they had histologically confirmed pancreatic ductal adenocarcinoma, locally advanced non-metastatic disease, and baseline ^18^F-FDG PET/CT imaging data available. They also had to be over 18 years old, have no history of malignancy within the past five years, and have sufficient follow-up imaging data to assess early progression. Patients were excluded if they had evidence of distant metastases at diagnosis, had received a diagnosis of another malignancy within the previous five years, were eligible for surgical resection, or had incomplete imaging datasets. All patients underwent a comprehensive baseline evaluation, which included clinical history assessment, performing a physical examination and carrying out laboratory tests (complete blood count, serum biochemistry and CA 19-9). Staging was performed using a multiphase, contrast-enhanced, thin-slice, multidetector CT scan optimized for pancreatic imaging. Diagnostic laparoscopy with peritoneal washing was performed when indicated. ^18^F-FDG PET/CT was used to complement staging and radiotherapy planning. Lymph nodes measuring less than 1 cm in diameter on CT were considered metastatic only if they demonstrated increased FDG uptake on PET. Tumor resectability was assessed by a multidisciplinary gastrointestinal tumor board according to the NCCN Guidelines (Version 2.2021), and all included patients were deemed to have unresectable LAPC.

This study was approved by the institutional ethics committee and conducted in accordance with the principles of the Declaration of Helsinki. Written informed consent was obtained from all participants. The protocols were registered at ClinicalTrials.gov with the identifiers NCT02984501 and NCT05399394.

### 2.2. Treatment Protocol

All patients received treatment in accordance with a therapeutic protocol involving induction chemotherapy followed by CRT. During the enrolment period, two chemotherapy regimens were used: 1000 mg/m^2^ of gemcitabine plus 100 mg/m^2^ of oxaliplatin, and the modified FOLFIRINOX scheme (oxaliplatin (85 mg/m^2^), irinotecan (180 mg/m^2^), leucovorin (400 mg/m^2^), and fluorouracil (2400 mg/m^2^)). Both regimens were administered every two weeks for a total of four cycles. Following induction chemotherapy, restaging was performed using contrast-enhanced CT imaging. Patients without radiological evidence of disease progression proceeded to receive CRT. Radiotherapy planning included four-dimensional CT simulation when possible, with strategies to manage respiratory motion applied to minimize internal organ displacement. The clinical target volume (CTV), defined as the primary tumor and involved lymph nodes, received a prescribed total dose of 59.4 Gy. Regional at-risk lymph node areas were treated with a doseof 45 Gy using standard fractionation. The planning target volume (PTV) was defined by expanding the CTV by an isotropic margin of 1 cm to compensate for setup uncertainties. CRT was delivered using a multi-leaf collimator and a multifield isocentric technique with linear accelerators (Varian Medical Systems, Palo Alto, CA, USA). Concurrent chemotherapy consisted of weekly administration of gemcitabine (600 mg/m^2^) during radiotherapy. Four weeks after completing the CRT, patients underwent clinical evaluation and a response assessment using CT imaging based on World Health Organization (WHO) criteria. Follow-ups were conducted every three months during the first two years and then every six months thereafter, in accordance with institutional protocols and the individual clinical course.

### 2.3. PET/CT Acquisition and Image Preprocessing

**Figure 1:**
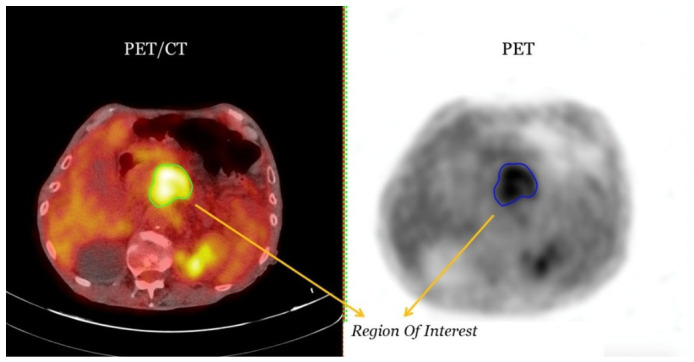
Transverse CT and PET scans of the same anatomical region. The segmented region of interest (ROI) can be seen in both images, enabling a direct visual comparison of anatomical and metabolic information.

All baseline ^18^F-FDG PET/CT scans were acquired at a single imaging center using a Biograph mCT scanner (Siemens Healthineers, Erlangen, Germany). Imaging was performed 60 ± 10 min after an intravenous injection of 236 ± 45 MBq of 18F-fluorodeoxyglucose (^18^F-FDG), with the administered activity adjusted according to each patient’s body mass index. The scan range extended from the skull base to the mid-thigh. Patients fasted for at least six hours prior to the scan, with serum glucose levels of less than 150 mg/dL at the time of tracer administration. All patients were administered 500 mL of intravenous saline to ensure adequate hydration. An initial X-ray scout scan was performed to define the anatomical field of view. This was followed by acquisition of a low-dose CT scan (120 kV, 50–80 mA) to correct for attenuation and localize the anatomy. PET acquisition was performed in 3D mode with a scan duration of two to three minutes per bed position. Image reconstruction was carried out using iterative algorithms in accordance with clinical standards. All scans were reviewed independently by two experienced nuclear medicine physicians. Each physician performed independent blinded assessments, and any discrepancies were resolved through a consensus discussion in order to establish the final labels. The readers were blinded to the clinical outcomes but were provided with all the baseline staging information. Areas of abnormal ^18^F-FDG uptake were defined as focal uptake that exceeded the background physiological distribution. In cases of disagreement, consensus was reached through joint review. Figure 1 shows transverse CT and PET images of the same anatomical region, with the segmented region of interest (ROI) clearly delineated. It should be noted that the CT component was acquired at a low dose and primarily intended for attenuation correction and anatomical localization. This may have limited the extraction of high-quality CT radiomic features relative to diagnostic contrast-enhanced CT.

A three-dimensional volume of interest (VOI) was manually delineated around the primary pancreatic tumor. Semi-quantitative PET metrics were then calculated within each VOI: SUVmax (defined as the maximum voxel value normalized to the injected dose and body weight) was measured using EQ·PET reference-based quantification (Siemens Healthineers). The metabolic tumor volume (MTV) was calculated using a 40% SUVmax threshold, and total lesion glycolysis (TLG) was obtained by determining the product of SUVmean and MTV. Throughout these steps, both the CT and PET scans had a color depth of 12 bits (2^12^), which provided enough intensity nuances to eliminate the need for further image enhancement. The only preprocessing step applied was image resizing, which was performed to ensure there were uniform dimensions across all scans and enhance feature comparability. Specifically, images were resampled to the median width and height observed across the dataset (this was performed only along the number of rows and columns while keeping the number of z-stack slices unaltered), with only a small proportion requiring adjustment.

**Figure 2:**
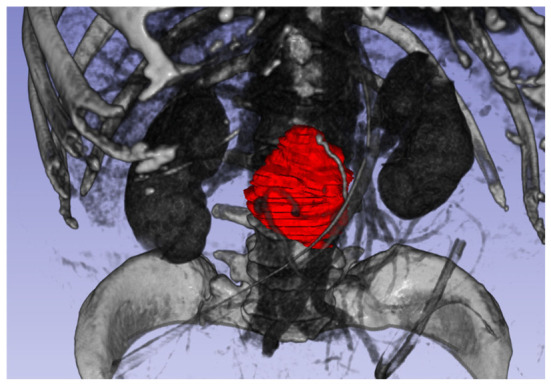
Three-dimensional representation of tumor volume (in red).

Figure 2 shows a three-dimensional rendering of the volume of interest (VOI) from the dataset.

### 2.4. Feature Extraction

Radiomic analysis was performed by extracting a comprehensive set of quantitative features from CT and PET images confined to the segmented gross tumor volume (GTV), which is hereafter referred to as the region of interest (ROI). To comprehensively characterize tumor phenotypes based on medical imaging, we computed three categories of features from the voxels within these ROIs: first-order features, derived from the voxel intensity histogram and describing basic statistical properties of the image signal; second-order GLCM features, which were extracted within a 3 × 3 × 3 cube that was centered on the reference voxel by evaluating all 26 three-dimensional directions at a distance of 1 voxel from [−1, −1, −1] to [1, 1, 1], without voxel resampling, based on the gray-level co-occurrence matrix to capture spatial relationships and texture patterns within the ROI; and second-order TOP-LBP features, obtained by applying the local binary pattern transform to encode local texture variations in voxel neighborhoods. Although local binary pattern on three orthogonal planes (LBP-TOP) was originally developed for spatiotemporal data, in this study, it was adapted to three-dimensional medical images by treating the volumetric stack (x, y, z) analogously to the (x, y, t) domain. In this framework, the third dimension is interpreted as an additional spatial axis rather than a temporal one. This approach enables the extraction of local texture patterns across three orthogonal planes, thereby enhancing the characterization of intratumoral heterogeneity. Similar extensions of local binary pattern methods to volumetric imaging have been reported in the literature [12,13,14]. Following feature extraction, missing or null values were addressed through a standardized harmonization process. Consequently, given the large number of extracted features, some values approached zero, leading to unreliable or non-computable derived features. Non-valid numbers (NaN values) were thus treated as missing values and imputed to minimize their impact on the feature table. Similar incompleteness arose from partial clinical data coverage, often due to real-world variations in diagnostic protocols, patients’ exclusion from certain tests, or retrospective data collection limitations. Missing continuous values were imputed with feature-wise means, as this approach preserves central tendency under approximate normality common in radiomic distributions. Discrete features used medians instead. This approach is more robust to outliers and better maintains the features’ non-continuous, ordinal nature. These strategies mitigated processing bias in subsequent pipeline steps. The resulting harmonized radiomic feature matrix was then integrated with the clinical dataset for each patient to create a unified data structure for subsequent analysis. All analyses were processed using pipelines developed in Python in the Google Colaboratory prototyping platform. Feature extraction and the methodological pipeline were implemented in Python 3.9, using NumPy, Pandas, and Scikit-learn. OsiriX was employed for 3D stack image analysis.

### 2.5. Radiomics Prediction Endpoint

The primary endpoint of this study was early progression (EP), which was selected to evaluate the ability of radiomic and clinical features to identify patients at high risk of treatment failure. EP was defined as radiologically confirmed local or distant disease progression at the first scheduled follow-up, approximately three months after treatment initiation. Importantly, this definition was applied uniformly across the entire cohort, regardless of whether patients proceeded to CRT or experienced progression after induction chemotherapy. We acknowledge that these subgroups represent distinct clinical trajectories; however, a unified definition was adopted to ensure consistency of the predictive endpoint across the dataset. In particular, in the patients who did not proceed to CRT, early progression reflects disease behavior during induction chemotherapy, which may differ from progression occurring after combined treatment. Progression of the disease was assessed using contrast-enhanced CT imaging and reviewed by experienced radiologists according to standard clinical criteria.

### 2.6. Machine Learning Pipeline

**Figure 3:**
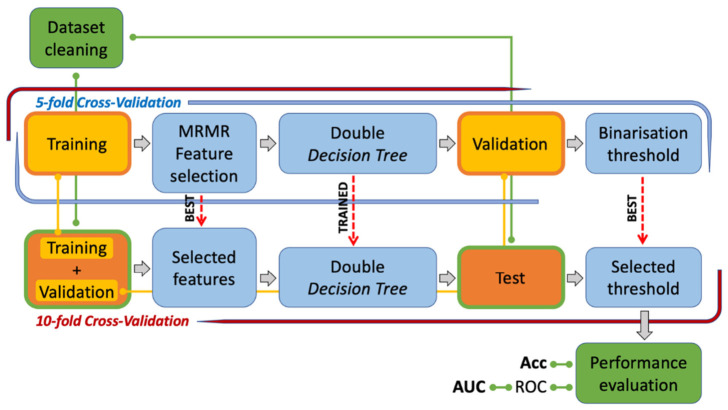
Radiomics pipeline.

The complete radiomics pipeline employed in this study is illustrated in Figure 3.

As previously described, the proposed approach began with the construction of the feature table, followed by a 10-fold cross-validation procedure (CV10). A 10-fold cross-validation strategy was employed to maximize training data utilization and simulate performance over a larger cohort despite the modest real-world size typical of LAPC studies. This approach ensured that all records contributed to both training and testing across folds, avoiding the exclusion risks of simple random splits. In contrast, leave-one-out CV was avoided due to its higher variance and instability due to output fluctuations, effects that are more pronounced in small datasets, while bootstrapping was deemed unnecessary as it introduces artificial data redundancy without proportional stability gains. In this procedure, the entire dataset was partitioned into ten equally sized folds, which were used iteratively to train and evaluate the pipeline and ultimately provided a robust performance estimate while ensuring the entire dataset served as the test set across folds, enabling prediction assessment for all records. The CV10 procedure comprised two distinct phases. First, a composite model was trained, and its optimal parameters were selected. Second, the trained and optimized model was evaluated on the test set for the current fold. During the first phase, after removing the records designated as the test set for the current iteration, we split the remaining data into two subsets: a training set with which to train the model and a validation set with which to select features without bias. To enhance robustness, we repeated this splitting process multiple times using an additional 5-fold cross-validation procedure (CV5) and then selecting the parameter setting that led to the best performance. Cross-validation splits were performed using stratified sampling to preserve the representation of the small early progression subset, thereby mitigating bias from imbalanced class distributions and enhancing result stability. Specifically, for each CV5 iteration, we used the Minimum Redundancy Maximum Relevance (MRMR) feature selection algorithm to identify the most relevant features. This algorithm selected features iteratively, balancing relevance and redundancy to produce a minimal yet highly predictive subset of features. We determined the optimal number of features by applying a scree plot criterion, evaluating feature importance scores to identify an inflection point beyond which additional features contribute only marginal improvements. After selecting the features, we trained a two-level decision tree (DT) model using the selected features. This model consisted of two DT classifiers that were trained using the same dataset but optimized using different false-negative (FN) cost parameters. Varying the FN costs meant that the first model adopted a less strict classification criterion to identify samples that exhibited clearer disease-related patterns. In contrast, the second model was more conservative and focused on detecting high-risk cases due to its higher FN cost. This complementary design leveraged the strengths of both classifiers to enhance predictive performance. During the validation and testing phases, each sample was initially evaluated by the low-FN-cost decision tree (DT). If the predicted class probability exceeded the 0.5 reliability threshold, the prediction was accepted as reliable. Otherwise, the sample was classified by the high-FN-cost DT, which prioritized sensitivity to risk and provided a secondary assessment for uncertain cases. Finally, computing the ROC curve using the validation set samples enabled us to determine the strictest classifier’s optimal operating point, fine-tuning its internal reliability threshold to achieve the best performance.

The lower part of Figure 3 shows the testing phase of the pipeline. During this phase, the two-level DT model was retrained using the combined training and validation sets, enabling it to fit a larger dataset. The hyperparameters were applied, as determined during the training and validation steps. Finally, we used the trained and optimized model to predict the samples in the test set, evaluating its performance using a confusion matrix (CM). The CM is a square matrix that compares predicted labels with true test labels, providing a compact visualization of the classifier’s performance. For a binary classification task, the CM is a two-by-two matrix where the main diagonal contains the number of correctly predicted true positives (TPs) and true negatives (TNs) and the off-diagonal elements represent the misclassified false positives (FPs) and false negatives (FNs), respectively. For the sake of completeness, we also calculated several secondary metrics: accuracy, the ROC curve, and the area under the curve (AUC).

### 2.7. Clinical Outcomes and Statistical Analysis

Descriptive statistics were used to summarize baseline demographic and clinical characteristics. Continuous variables were reported as the mean or median (range), depending on the data distribution, while categorical variables were expressed as absolute frequencies and percentages. Overall survival (OS) was defined as the time from histological diagnosis to death by any cause or, for censored cases, to the date of the last follow-up. Progression-free survival (PFS) was calculated from the start of treatment until documented disease progression (local or distant) or death, whichever occurred first, with censoring at the last follow-up if the event did not occur. Local progression-free survival (LPFS) was defined as the interval from treatment initiation to local progression at the primary tumor site, as assessed according to RECIST criteria. It was censored at the last follow-up if no local events were observed. Metastasis-free survival (MFS) was calculated from the start of treatment to the detection of distant metastases, with censoring at the last follow-up in the absence of metastatic progression. As previously detailed, early progression was the primary endpoint of the radiomics analysis. It was defined as radiologically confirmed local or distant progression at the first scheduled follow-up. This was performed approximately three months after treatment initiation and assessed using contrast-enhanced CT imaging. In addition to serving as the target outcome for predictive modelling, the incidence of EP in the study cohort was evaluated. Survival curves for OS, PFS, LPFS and MFS were generated using the Kaplan–Meier method where applicable. All statistical analyses were performed using the Statistical Package for the Social Sciences (SPSS) version 29.0 (IBM Corp., Armonk, NY, USA).

## 3. Results

### 3.1. Patients

This study included 57 patients, with a median age of 64 years (range: 40–79). Slightly more than half of the patients were women (54.4%). Most tumors were located in the pancreatic head (92.9%), with only 7.1% located in the body or tail. The median baseline CA 19-9 level was 1044 U/mL (range: 1–3848). At diagnosis, 28.1% of the patients were classified as borderline resectable, while 71.9% were unresectable. Regarding the pre-treatment ^18^F-FDG PET/CT scan, the mean time interval between the scan and the start of treatment was 16 days (range, 2–40 days). The mean SUVmax, MTV, and TLG values for primary tumors were 6.7, 20.6 cc, and 88.4, respectively. All patients completed the induction chemotherapy phase, with 34 (59.6%) proceeding to CRT. Table 1 summarizes the baseline demographic, clinical and tumor characteristics of the study cohort.

### 3.2. Clinical Outcomes

The median follow-up for the entire cohort was 12.8 months (range, 2.6–92.9 months). At the time of the last evaluation, 16 patients were alive. One patient underwent restaging earlier than scheduled due to clinical suspicion of progression. Early progression at the first evaluation was observed in 20 patients (35%): 2 had local progression only, 12 had distant metastases only, and 6 presented with both local and distant progression. Overall, 38 patients (66.6%) experienced disease progression during the follow-up. Of these patients, four experienced local progression only, while 34 developed distant metastases, including 11 patients who also had local progression.

**Figure 4:**
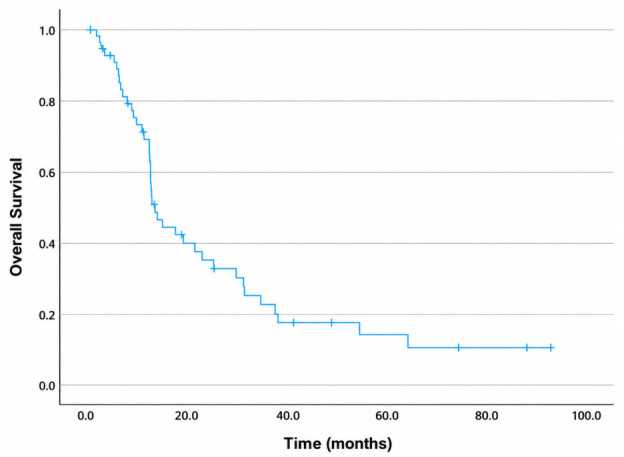
Kaplan–Meier curve for overall survival.

**Figure 5:**
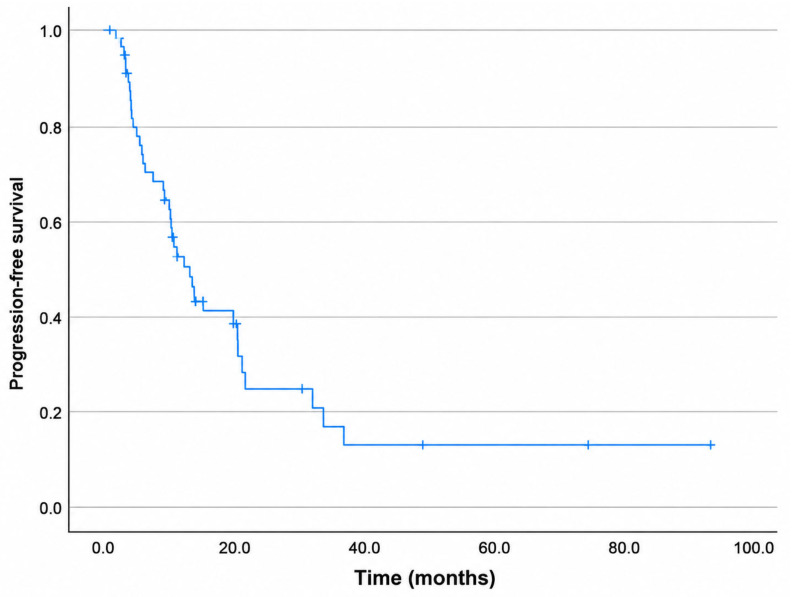
Kaplan–Meier curve for progression-free survival.

**Figure 6:**
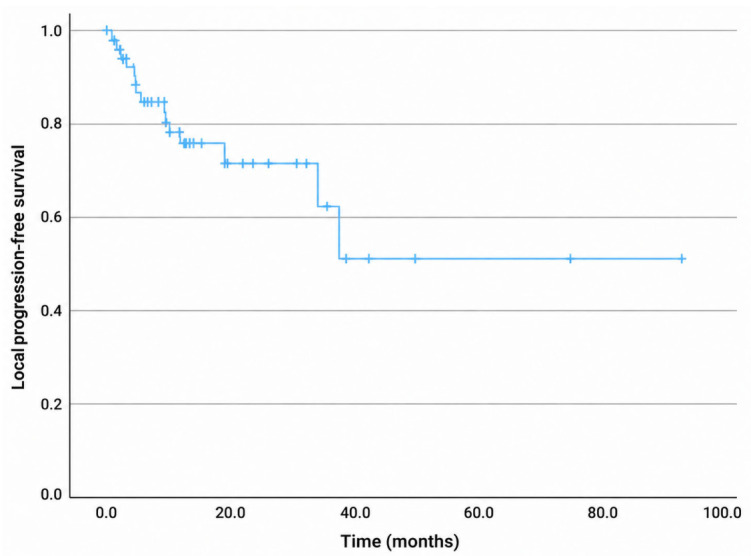
Kaplan–Meier curve for local progression-free survival.

**Figure 7:**
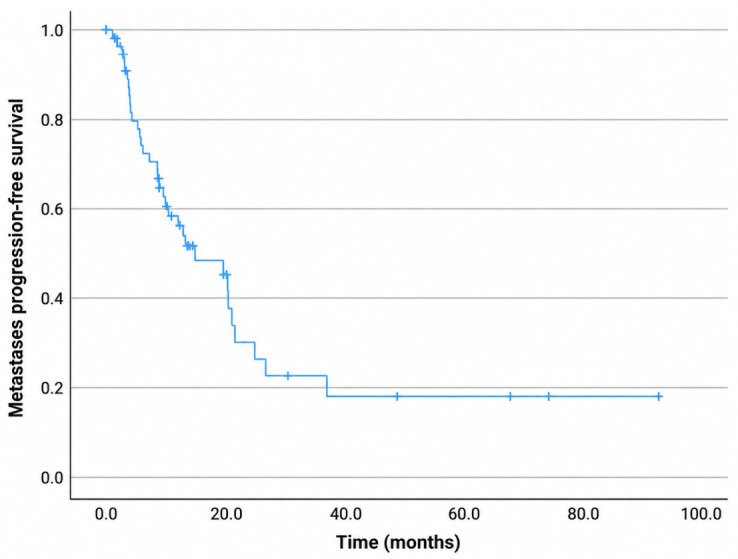
Kaplan–Meier curve for metastases progression-free survival.

For the entire cohort, the median OS was 13.4 months, with 1-, 2-, and 3-year OS rates of 70%, 35%, and 22%, respectively (Figure 4). The median PFS was 11 months, with 1-, 2-, and 3-year PFS rates of 48%, 24%, and 12%, respectively (Figure 5). The median LPFS was not reached; the 1-, 2-, and 3-year LPFS rates were 75%, 71%, and 52%, respectively (Figure 6). The median MFS was 12.2 months, with 1-, 2-, and 3-year MFS rates of 51.6%, 26.3%, and 18%, respectively (Figure 7).

### 3.3. Radiomic Model Performance

**Figure 8:**
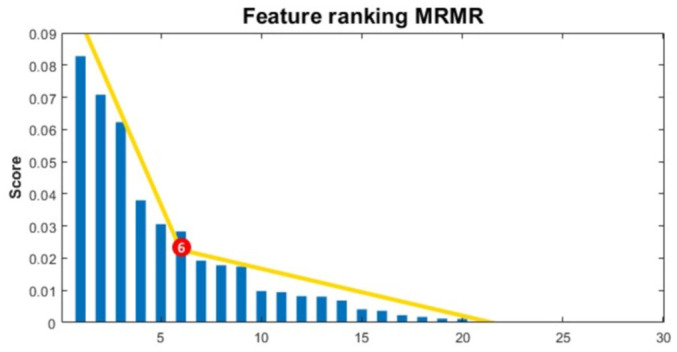
Minimum redundancy–maximum relevance and scree plot for feature selection.

A total of 242 radiomic features were extracted from each imaging modality (CT and PET), including 12 first-order, 182 GLCM, and 48 LBP-TOP features. These were then combined with PET metrics and clinical variables. Figure 8 shows the results of the feature selection process for one CV5 fold. The corresponding scree plot illustrates the number of features selected. This process ensured that only the most informative and non-redundant features were retained for training the model. Due to the cross-validation design, feature selection was performed independently within each fold, resulting in variability in the selected feature subsets. To improve transparency, Supplementary Table S1 reports representative feature classes and candidate variables contributing to the multimodal model, together with their modality, biological rationale, and approximate selection frequency ranges derived from repeated cross-validation analyses.

**Figure 9:**
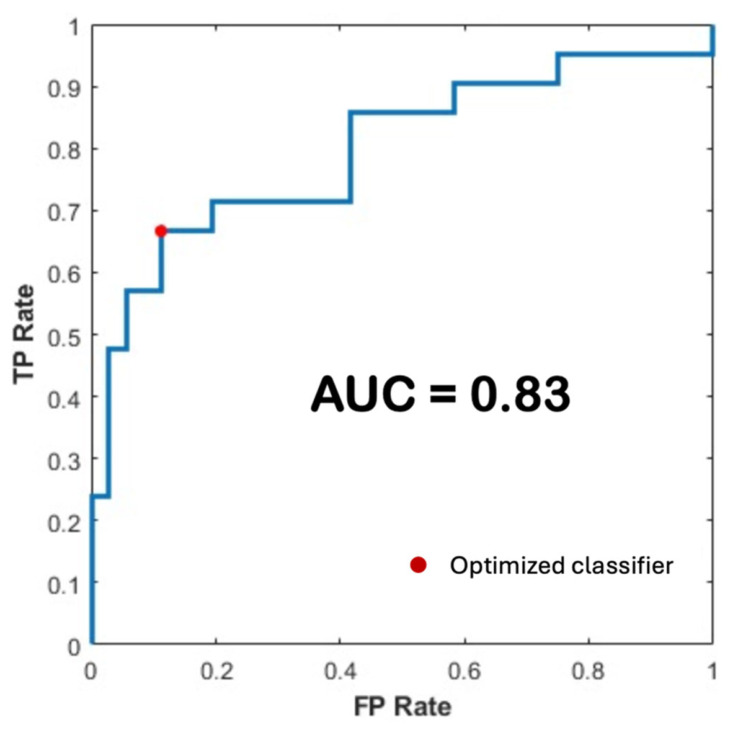
ROC curve of the test set predictions, with the operating point of the optimized classifier indicated in red.

On the independent test set, the proposed pipeline achieved an accuracy of 80.7% and an area under the curve (AUC) of 83%, demonstrating its robust predictive performance. Moreover, the 10-fold cross-validation experiments yielded a confidence interval width of less than 0.1. Figure 9 shows the ROC curve of the optimized model, confirming its strong discriminative ability.

**Figure 10:**
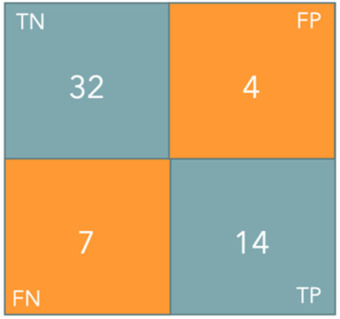
Confusion matrix of the test set predictions.

Figure 10 shows the confusion matrix, which illustrates the distribution of predictions across the two classes and highlights balanced classification performance.

## 4. Discussion

Our study demonstrates that baseline ^18^F-FDG PET/CT radiomic features, when combined with clinical and biological data, can accurately predict early progression in patients with LAPC. Using a two-level decision tree model with optimized feature selection, we achieved an accuracy of 80.7% and an AUC of 0.83 on independent test data. These results suggest that high-risk patients with aggressive disease phenotypes can be recognized prior to treatment. For the sake of completeness, prior to early fusion of imaging, we conducted ablation experiments evaluating feature families individually using CT radiomic features alone, clinical variables alone, and PET metrics alone. Single-modality models consistently performed worse than the integrated multiparametric approach, likely due to incomplete capturing of tumor heterogeneity and clinical context. Consequently, only the results of the fused model are reported, emphasizing the importance of combining imaging biomarkers with clinical data for reliable early prediction of progression in LAPC. In our cohort, 20 out of 57 patients (35%) experienced disease progression within approximately three months of therapy. This highlights the importance of identifying these patients early on so that therapeutic intensity can be guided and unnecessary toxicity avoided. The high accuracy and balanced sensitivity of our model, as indicated by the confusion matrix, suggest that integrating high-dimensional imaging features with clinical markers can capture tumor aggressiveness in a way that goes beyond conventional metrics. In this context, to avoid redundancy bias commonly introduced by broader feature sets (e.g., extensive IBSI-compliant extractions yielding highly correlated GLCM variants), we pre-tested and selected a focused combination of first-order, GLCM, and LBP-TOP features. This targeted approach minimized collinearity, simplified subsequent feature selection, and enhanced model interpretability without sacrificing predictive power [15]. Our findings extend the preliminary observations made in our previous analysis [8], in which conventional PET-derived metrics (MTV and TLG) and CA 19-9 were identified as predictors of early progression and survival. Building upon this foundation, the present study leverages radiomic texture features to capture intratumoral heterogeneity beyond traditional PET parameters, thereby enhancing predictive performance. This methodological advancement is consistent with previous findings that radiomic signatures outperform single-parameter metrics, such as SUVmax or MTV. Toyama et al. found that texture-derived features, particularly the gray-level zone length matrix non-uniformity (GLZLM GLNU) measure, were the strongest predictors of survival, surpassing SUVmax and total lesion glycolysis (TLG). Their study demonstrated the added value of textural heterogeneity measures by showing that combining GLZLM GLNU with TLG enabled patients to be stratified into distinct prognostic groups [16]. Similarly, Kang et al. reported that a radiomic risk score derived from PET/CT features significantly improved the prediction of overall survival over a clinical model alone (with a combined C-index 0.74 vs. 0.67) [17]. Thus, our results reinforce the idea that quantitative imaging biomarkers capture biological aggressiveness in PDAC. In particular, the high AUC for EP in our study suggests that radiomics can detect subtle patterns linked to early metastatic or local spread. While conventional PET-derived metrics have demonstrated prognostic value, they provide only limited information on tumor heterogeneity. In contrast, radiomic features capture complex spatial patterns and relationships within a tumor, enabling a more comprehensive characterization of tumor phenotype. This idea was supported in our study by the ablation analyses, where single-modality models consistently underperformed compared to the integrated approach. These observations are also supported by wider radiomics research into pancreatic disease. A systematic review has noted that radiomic features, particularly second-order (texture) statistics, often provide valuable information about prognosis or treatment response in regard to pancreatic lesions [18]. Abunahel et al. concluded that radiomics holds promise as a quantitative imaging biomarker in pancreatic cancer but emphasized that the usefulness of features varies by application [18]. Similarly, Bartoli et al. (2020) highlighted that CT/MRI-based radiomics analyses are increasingly being used to explore tumor heterogeneity and the tumor microenvironment in PDAC [19]. Our use of FDG-PET/CT radiomics is in line with these advances, given that PET can more directly reflect tumor metabolism and hypoxic patterns. In LAPC, where tumor biology drives rapid dissemination, PET-derived heterogeneity measures may therefore be particularly prognostic. Notably, our pipeline capitalized on this by analyzing CT and PET texture in a unified way, an approach that, to our knowledge, has not been applied to the prediction of early progression in regard to pancreatic cancer. From a translational perspective, these findings could have implications for personalized therapy. EP reflects an aggressive disease phenotype with poor outcomes. Identifying high-risk patients early on could allow clinicians to tailor treatment intensity, enroll patients in clinical trials of novel systemic agents, or prioritize palliative approaches in selected cases. Conversely, patients predicted to be low-risk could safely proceed with locoregional treatments or surgical exploration. This approach aligns with the vision of precision oncology, in which imaging-derived biomarkers complement clinical and molecular tools, refining patient stratification and therapeutic decision-making.

Despite the promising results, there are several limitations that warrant discussion. Firstly, the sample size of our study was relatively small (N = 57), and the patients were recruited from a single center, which may limit generalizability and raise the risk of overfitting. The relatively small sample size and absence of external validation may have limited the generalizability of our findings and led to optimistic performance estimates. To mitigate this issue, we adopted a nested cross-validation strategy, combining an outer 10-fold cross-validation with an inner 5-fold cross-validation for model selection and hyperparameter tuning. This approach allows efficient use of the available data while reducing selection bias and providing a more reliable estimate of model performance compared to a single hold-out split. Previous methodological studies have shown that nested or repeated cross-validation schemes are preferable in small-sample settings, as they better account for model-selection uncertainty and reduce overfitting, although some degree of optimism may still remain [20,21]. Therefore, the present results should be considered exploratory and require confirmation in larger, independent cohorts. Secondly, our analysis was retrospective. Thirdly, we focused on baseline imaging and did not consider dynamic changes during therapy. Mid-treatment imaging could enhance prediction and is a promising area for future research, as suggested by Wang et al. [22]. At the same time, Malcolm et al. have noted that PDAC radiomics studies often suffer from substantial methodological heterogeneity and limited reproducibility due to differences in imaging protocols [23]. Although we applied cross-validation and rigorous feature selection and standardized our pipeline to address these issues, prospective validation in larger multicenter cohorts will be essential to confirm its robustness. Another limitation is our use of low-dose CT for radiomic feature extraction, which may have reduced the quality and reproducibility of CT-derived features relative to diagnostic contrast-enhanced imaging. A direct comparison between radiomic-derived heterogeneity and visual assessment by expert readers was not performed in this study. Further research integrating qualitative and quantitative imaging evaluation could help clarify their relative contributions and potential complementarity. Moreover, the model achieved a sensitivity of 66.7%, indicating that approximately one-third of early progression cases were not identified. In this clinical context, this limitation is relevant, as missing high-risk patients could affect treatment decisions and clinical outcomes. Therefore, at its current stage, the model should be considered a supportive tool rather than a standalone decision-making system. On the other hand, the relatively high specificity (88.8%) suggests a low false-positive rate, which may help avoid unnecessary treatment modifications. This reflects an inherent trade-off between sensitivity and specificity that could be further optimized in future studies. Our study also has several methodological strengths. Unlike many retrospective radiomics studies, our cohort was derived from two prospective clinical protocols, which ensured standardized staging, treatment, and follow-up. This design reduces heterogeneity and enhances the reliability of our results. Furthermore, all FDG-PET/CT scans were acquired in a single specialized imaging center under consistent protocols, thereby minimizing variability due to differences in scanners or radiotracer administration. This uniformity improves the reproducibility of radiomic feature extraction and represents a unique strength relative to multi-institutional datasets with variable imaging practices. The potential application of radiomics to FAPI PET imaging represents an interesting future direction. Given its higher tumor contrast and reduced background activity, FAPI-based radiomics may further improve feature robustness and predictive performance, although this remains to be validated.

## 5. Conclusions

We developed and evaluated a multiparametric predictive model integrating baseline PET/CT radiomic features with clinical and biological data to identify LAPC patients at high risk of early progression. The model showed promising predictive performance and added value over traditional metrics. These findings highlight the potential of imaging-based biomarkers in precision oncology for pancreatic cancer, as radiomics can non-invasively capture tumor heterogeneity and support individualized treatment planning. Our findings should be interpreted as preliminary and considered exploratory and hypothesis-generating, warranting confirmation in larger, multicenter cohorts. If validated in larger cohorts, our approach could assist clinicians in tailoring therapy intensity or selecting candidates for innovative clinical trials, ultimately improving patient management. Future research should prioritize external validation, integration with complementary biomarkers, and prospective evaluation of radiomics-guided treatment strategies.

## Figures and Tables

**Table 1:** Patients’ characteristics.

*Characteristics*	*No Patients (All = 57)*	*%*
*Age (yrs)*		
Median	64	
Range	40–79	
*Sex*		
Male	26	45.6
Female	31	54.4
*Location*		
Head	53	92.9
Body/Tail	4	7.1
*Ca19.9 (U/mL)*		
Median	1044	
Range	1–3848	
*Resectability status*		
Borderline	16	28.1
Unresectable	41	71.9
*Baseline SUVmax*		
Mean	6.7	
Range	1.1–21.1	
*Baseline MTV (cc)*		
Mean	20.6	
Range	3.3–82.9	
*Baseline TLG*		
Mean	88.4	
Range	9.6–443.9	
*Induction Chemotherapy*		
Yes	57	100
No	0	0
*Concurrent chemoradiation*		
Yes	34	59.6
No	23	40.4

SUV: standardized uptake value; MTV: metabolic tumor volume; TLG: total lesion glycolysis.

## Data Availability

The original contributions presented in this study are included in the article. Further inquiries can be directed to the corresponding author.

## Supplementary Materials

The following supporting information can be downloaded at https://www.mdpi.com/article/10.3390/diagnostics16101499/s1. Table S1: Representative feature classes and candidate variables contributing to the multimodal predictive model, grouped by modality and feature family.
