# Supplementary material for: Exploratory PET/CT Radiomics for Predicting Early Progression in Locally Advanced Pancreatic Cancer
Source: Diagnostics (Basel). 2026 May 14;16(10):1499. doi: 10.3390/diagnostics16101499 (PMC13205166; doi:10.3390/diagnostics16101499)
Supplement: Supplementary file 1 [file diagnostics-16-01499-s001.zip › diagnostics-4260104-supplementary.pdf]

Table S1. Representative feature classes and candidate variables contributing to the multimodal predictive model, grouped by modality and feature family.

Feature / Variable	Modality	Feature Family	Description / Biological Rationale	Selection frequency
CA 19-9	Clinical	Clinical/Biochemical	Established biomarker associated with tumor burden and prognosis in pancreatic cancer	88-100%
Resectability status	Clinical	Clinical	Reflects baseline anatomical extent and vascular involvement	75-95%
Clinical stage at diagnosis	Clinical	Clinical	Reflects disease extent at baseline and is associated with prognosis and treatment strategy	78-95%
Age	Clinical	Clinical	General prognostic factor in oncology	10-30%
SUVmax	PET	Conventional PET metric	Reflects maximum metabolic activity within the tumor	55-72%
MTV	PET	Conventional PET metric	Quantifies metabolically active tumor volume	47-66%
TLG	PET	Conventional PET metric	Combines metabolic activity and tumor volume	45-65%
First-order intensity features (e.g., mean, entropy)	PET	First-order	Describe voxel intensity distribution and metabolic heterogeneity	35-56%
First-order intensity features (e.g., mean, skewness)	CT	First-order	Capture density distribution within the tumor	46-65%
GLCM texture features (e.g., contrast, entropy, homogeneity)	PET	Second-order texture	Characterize spatial relationships of metabolic uptake	29-44%
GLCM texture features (e.g., contrast, correlation)	CT	Second-order texture	Reflect structural heterogeneity of tumor tissue	34-55%
LBP-TOP features (e.g., energy, entropy)	PET	Higher-order texture	Capture local multi-planar patterns of metabolic heterogeneity	87-100%
LBP-TOP features (e.g., uniformity, variance)	CT	Higher-order texture	Describe fine-scale spatial variations in tissue structure	90-100%

As shown in Table S1, feature selection across repeated cross-validation iterations revealed consistent patterns. Higher-order texture features, particularly LBP-TOP descriptors from both PET and CT, were among the most frequently selected variables, together with key clinical factors such as CA 19-9 and resectability status.

PET-derived volumetric parameters (SUVmax, MTV, TLG) showed intermediate selection frequencies, reflecting their complementary contribution to metabolic characterization. In contrast, first-order and GLCM features exhibited more variable selection patterns, likely due to redundancy within larger feature families.

Overall, these findings suggest that the model preferentially relies on texture-based descriptors and clinically relevant variables to capture tumor heterogeneity and predict early progression.
